# Data obtained with an open-source static automated perimetry test of the full visual field in healthy adults

**DOI:** 10.1016/j.dib.2018.09.079

**Published:** 2018-09-29

**Authors:** Iván Marín-Franch, Paul H. Artes, Luke X. Chong, Andrew Turpin, Michael Wall

**Affiliations:** a Department of Ophthalmology and Visual Sciences, University of Alabama at Birmingham School of Medicine, Birmingham, AL, USA; b Eye & Vision Research Group, Institute of Health and Community, Plymouth University, UK; c School of Medicine, Deakin University, Geelong, Australia; d School of Computing and Information Systems, University of Melbourne, Australia; e Departments of Neurology and Ophthalmology and Visual Sciences, University of Iowa, College of Medicine, Iowa City, IA, USA; f Iowa City Veterans Administration Health Care System, IA, USA

## Abstract

The data were gathered from 98 eyes of 98 ocular healthy subjects. The subject ages ranged from 18 to 79 years with a mean (and standard deviation) of 47 (17) years. Each subject underwent two visual field tests, one of the central visual field (64 locations within 26° of fixation) and one of the peripheral visual field (64 locations with eccentricity from 26° to up to 81°). Luminance thresholds for the Goldmann size V stimulus (with a diameter of 1.72° of visual angle) were obtained with the ZEST Bayesian test procedure. Each test was conducted twice within 90 days.

## Specifications table

**Table:** 

Subject area	*Clinical vision science*
More specific subject area	*Perimetry*
Type of data	*Excel file*
How data was acquired	*Testing on an Octopus 900 commercial perimeter driven by the Open Perimetry Interface (OPI), an open-source R tool for designing and conducting perimetry at custom locations and with custom methods and algorithms*
Data format	*Raw, filtered, and analyzed*
Experimental factors	*Visual stimuli presentation is made following a random sequence of spatial locations. At each location, luminance threshold are determined following the Bayesian test procedures of King-Smith et al. (ZEST algorithm)*
Experimental features	*Volunteers tested for on the OPI-driven Octopus 900 using a larger stimulus size (Goldmann size V) than in conventional perimetry*
Data source location	*Iowa City, Iowa, USA*
Data accessibility	*Data is in this article and in the open source R package visualFields.*
Related research abstract (ARVO abstract)	*E. Lee, A. Subramani, R. Wanzek, T. Eden, L. X. Chong; A. Turpin; I. Marín-Franch, and M. Wall, Patterns of Vision Loss in Idiopathic Intracranial Hypertension: The Central vs. Peripheral Visual Field. Invest. Ophthalmol. Vis. Sci. 58 (2017) 3314*[10]

## Value of the data

• This is the first attempt to examine the central and peripheral visual field of a group of healthy individuals with an open-source threshold automated visual field test implemented in the Open Perimetry Interface.
• This test uses a Goldmann stimulus size V that has better retest variability, greater useful dynamic range and greater saliency in the far periphery than the Goldmann size III stimulus.
• This data is useful for clinical researchers to design tests and analyses of the far peripheral visual field.
• Each subject underwent each test twice so that retest variability can be quantified.

## 1 Data

**Fig. 1:**
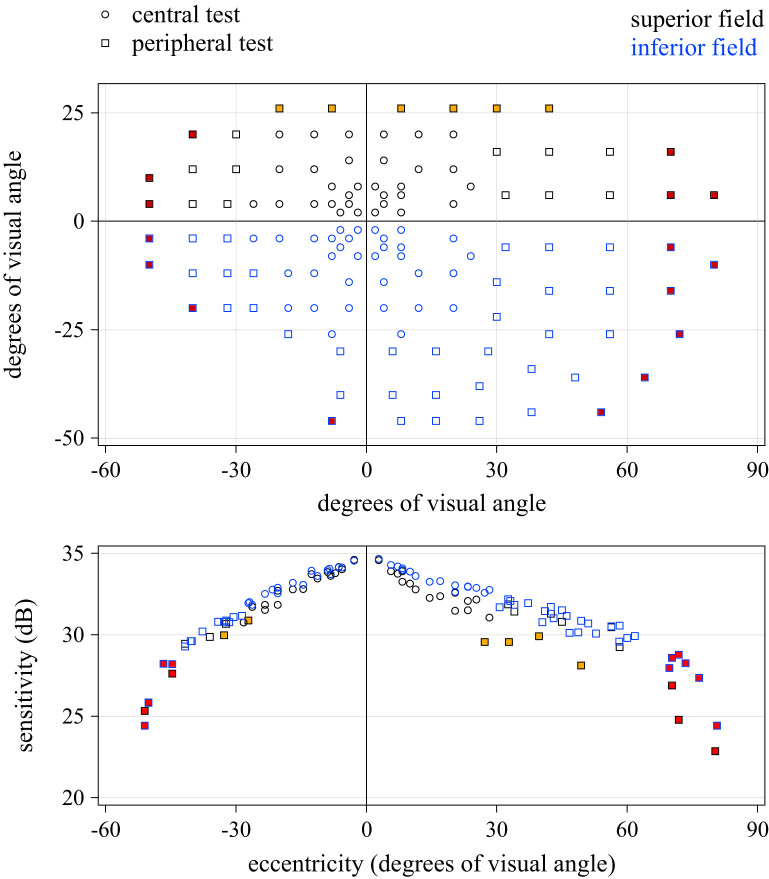
The upper panel shows the locations of the visual fields test locations for the central 30° (circles) and the peripheral (squares) perimetry tests. The lower panel shows the mean normal values for a 50-year-old subject as a function of eccentricity from the fovea. Locations in the superior part of the visual field are shown in black and locations in the inferior part in blue. The symbols filled in red correspond to locations with mean normal sensitivity lower than 29 dB. The symbols filled in yellow correspond to locations that are typically affected by eyelid artifacts.

The dataset consists of 128 luminance thresholds, obtained at 64 locations within the central 26° of the visual field and at 64 locations from 26° to up to 81° of the visual field for 98 eyes of 98 subjects, each eye tested twice. Put together, the central and peripheral tests cover from − 50° to 80° of the visual field horizontally and from − 46° to 26° vertically, that is, the full visual field (in a clinically useful sense). The precise locations tested are shown in the upper panel of Fig. 1. Although the visual field expands beyond 26° vertically, the upper eyelid is very often in the way (creating what is known as upper eyelid artifacts) so that the locations at 26° and farther up appear to have depressed sensitivity. Those locations are of limited clinical usefulness. For each test, the data include the eye tested, the subjects׳s age, proportion of false positives and false negatives, test duration and pauses, number of presentations, and the sensitivity estimated at each location.

## 2 Experimental design, materials and methods

### 2.1 Subjects

One hundred ocular healthy subjects, approximately 10 per decade, were each tested twice within 90 days. The subjects had an average age of 47 years, with a standard deviation of 17 years and ranging from 18 to 79. Seventy-one of the subjects were women and 27 were men. The numbers of subjects per decade are shown in Table 1. Subjects were considered ocularly healthy if they had no history of eye disease and refractive error of less than 5 diopters (D) of sphere and 4 D astigmatism, no history of diabetes mellitus or systemic arterial hypertension, and a normal ophthalmologic examination including 20/25 or better best-corrected Snellen visual acuity. The subjects either had undergone a complete eye exam within 12 months prior to this study or were examined by an ophthalmologist on the day of testing to ensure normal ocular health. They all passed a general health questionnaire. All subjects underwent a preliminary conventional static automated perimetry test. Subjects that did not meet the reliability criteria of < 15% false positive and false negative rates were not included in the study. Two subjects were removed from the final dataset, one because the subject withdrew and there is no retest information and a second because of a breach of testing protocol. The visual testing protocol was approved by the University of Iowa Institutional Review Board. The study followed the tenets of the Declaration of Helsinki. The subjects answered advertisements inviting them to participate in research and were paid in agreement with the Institutional Review Board. All subjects gave written informed consent to participate in the study.

**Table 1:** Sample size, *n*, per decade.

Age	*n*
18–20	2
20–29	17
30–39	17
40–49	15
50–59	18
60–69	14
70–79	15
Total	98

### 2.2 Experimental protocols

At the first visit, an eye was chosen at random. The central visual field was tested first and the peripheral field second. For the central test, refractive correction was applied as usual in a clinical practice. For the peripheral test, there was no eye correction because the frame of the corrective lens would generate a lens rim artifact (see, e.g., [1]) limiting the extend of the visual field that can be tested. No practice test was given.

An Octopus 900 commercial device was used and operated using the Open Perimetry Interface (OPI) [2]. The Bayesian test procedure ZEST [3], [4] was used to estimate luminance thresholds at each location. The background luminance was set at 10 cd m^−2^, as in conventional perimetry, and the maximum brightness was 1273 cd m^−2^ (4000 apostilbs). For each location, the prior probability mass function was bimodal with one peak at 15 dB. The second peak was set at 33 dB for 4 seed locations located at coordinates (± 8°, ± 8°) in the central test and 31 dB for 7 seed locations in the peripheral test (coordinates shown in Table 2). These values are the average sensitivities for healthy eyes obtained from a preliminary study. For all other locations, the sensitivity of the second peak was calculated during runtime by taking the mean sensitivity of its direct neighbors. The bimodal prior implemented here was based on a probability density function described in [5]. The test domain was from 10 dB to 45 dB. The procedure was truncated at 15 dB and estimated sensitivities below 15 dB were censored and assigned the value − 1. After each presentation, the probability mass function was updated depending on the subject׳s response until the estimated standard deviation was less than 1.5 dB. The estimated threshold was determined by calculating the mean of the posterior probability mass function upon termination. Details about the algorithm can be found elsewhere [6], and are also implemented in the R package OPI [2]. The code based on the OPI package used to collect this dataset can be found in the github repository https://github.com/lxchong/perimetry-custom-grids.

**Table 2:** Coordinates of primary seed locations of the peripheral visual field test.

*x*	*y*
30	16
− 30	12
56	6
− 32	− 12
70	− 16
28	− 30
64	− 36

A maximum of 14 false positive and 14 false negative catch trials were presented throughout the test. Ten catch trials were within the first 60 stimulus presentations and one every 20 thereafter. For false positive trials, a 55 dB stimulus was presented to the subject. For false negative trials, the algorithm added 10 dB of brightness to a test location that had been seen with a luminance corresponding to at least 20 dB of sensitivity. Responses faster than 150 ms were flagged as being too fast and subject responses slower than 600 ms were flagged as being slow. The technician monitored fixation throughout the duration of the test.

A feature of these testing procedures that depart from conventional perimetry is a reduction of the dynamic range to stimuli with no more than 15 dB of attenuation since these values fall outside the useful dynamic range of perimetric stimuli [7].

## 3 Statistical summary of dataset

Fig. 1 shows the test locations for the central and peripheral visual field tests. As it is customary in visual psychophysics, the luminance thresholds were converted into sensitivity (defined as the inverse of threshold) and expressed in decibels (dB) of attenuation.

The upper panel of Fig. 1 shows the locations for the central 30° (circles) and the peripheral (squares) perimetry tests and the lower panel shows the expected mean sensitivity values in dB for a 50 year-old subject as a function of eccentricity.

**Fig. 2:**
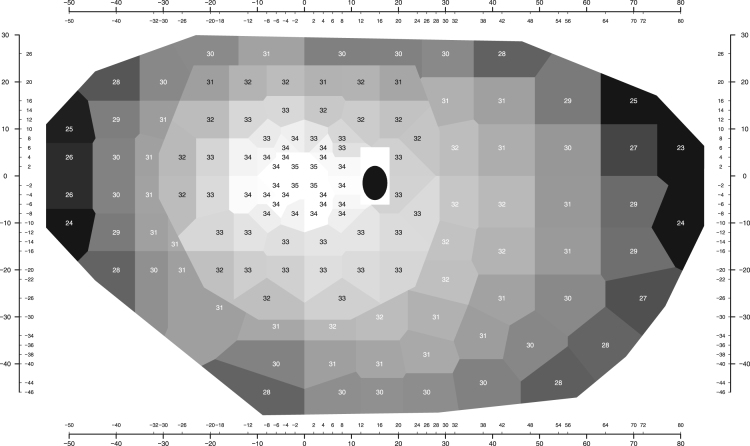
Mean normal values for each location. The rectangular region in white around (15°, − 3°) and (15°, 3°) correspond to locations that are typically excluded from analysis because of their proximity to the anatomical blind spot (represented here by a black ellipse). They were not included in this custom-made grid of test location (see Fig. 1). Data for the central test is shown in black and data for the peripheral test in white. Some locations in the edge between the central and peripheral tests were moved slightly for graphical clarity.

**Fig. 3:**
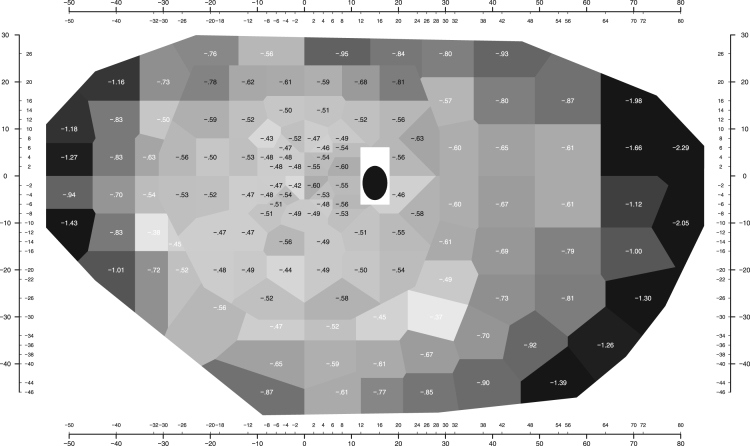
Age effect: slopes (decrease in mean normal sensitivity in dB per decade) for each location. Other details as for Fig. 2.

Fig. 2 shows the mean normal values for a 50 year-old subject for each test location and Fig. 3 shows the age effect (decrease in mean normal sensitivities in dB per decade). For better graphical visualization, the Voronoi tessellation [8] was used to delimit the regions for each test location.

**Fig. 4:**
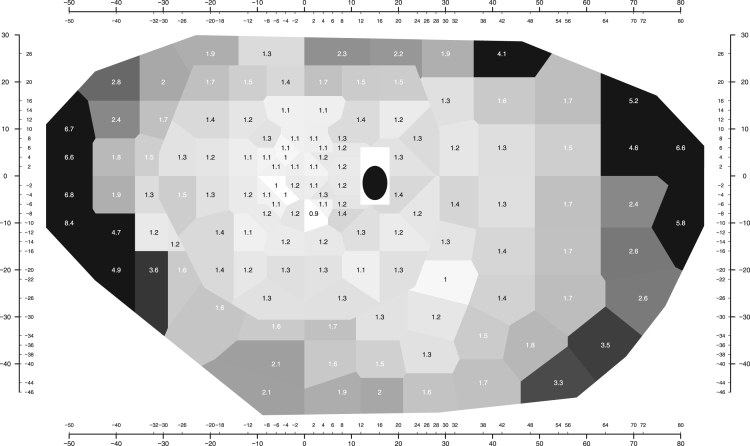
Standard deviation of total deviation values. In this figure, the text is shown in black or white only to increase contrast with the background gray scale (unlike in Fig. 2). Other details as for Fig. 2.

**Fig. 5:**
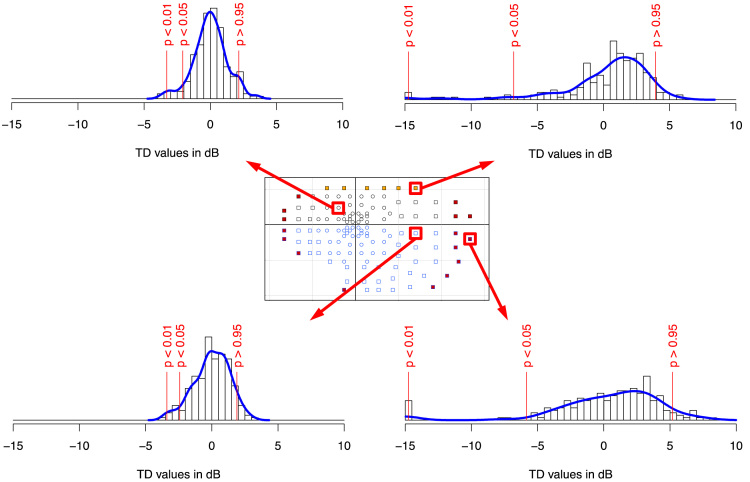
Histograms of total deviation values in four locations of the visual field. The four histograms are presented for the same scale for relative frequencies. The vertical red lines show the empirical quantiles 0.01, 0.05, and 0.95. Top left panel shows data for a location from the central 26° perimetry test. Bottom left panel shows data for a location in the peripheral test. Upper right panel shows data for a location that is susceptible to eyelid artifacts. Bottom right panel shows one of the two locations that are furthest away from the fovea.

For interpretation of visual fields, it is common to remove age-related visual losses from the data by subtracting the expected normal values for a subject of the same age from the observed sensitivities. The resulting differences are called total deviation [9]. A negative total deviation value means lower sensitivity than normal, whereas a positive total deviation value means greater sensitivity than normal. Fig. 4 shows the pointwise standard deviations of the total deviation values. Fig. 5 shows the distribution of total deviation values for four locations.
